# OP19 Multiple-Criteria Decision Analysis: Techniques To Support Environmental Sustainability Framework Development In Health Technology Assessment

**DOI:** 10.1017/S0266462324000825

**Published:** 2025-01-07

**Authors:** Melissa Pegg, Janet Boutell, Matthew Taylor

## Abstract

**Introduction:**

Technical and trade-off issues to incorporating environmental data in health technology assessment (HTA) decision processes are known. Multiple-criteria decision analysis (MCDA) has been successfully used across disciplines, supporting multifaceted technology decision-making. Challenges in prioritizing environmental criteria, resource constraints, and other criteria to assess technologies can be overcome through the application of MCDA, also used in conjunction with other techniques in HTA.

**Methods:**

A systematic review of methods to evaluate environmental sustainability in HTA was conducted following the PRISMA guidelines. Using a comprehensive search strategy, Ovid Embase, Web of Science (all databases), PubMed, EBSCOhost GreenFILE, IEEE Xplore, international HTA database, Cochrane Library, and grey literature were searched. The review analyzed the following broad themes: methods to overcome barriers, including how methods can handle “trade-off” issues between financial and environmental considerations; resource and expertise constraints; and data quality issues. This review determined how comprehensive the methods are for assessing sustainability in HTA. Frameworks were also ranked based on their overall transparency and feasibility.

**Results:**

This review (SR) identified 10 key studies. Half of these studies outline MCDA models within the frameworks. All MCDA studies have been combined with other techniques to support sustainable technology decision-making HTA. Analytical MCDA models with roots in mathematics are highlighted as reproducible techniques to support multifaceted decision-making, particularly where there are conflicting criteria. Analytical hierarchy process (AHP) used in conjunction with a circular economy framework for health technology supports global health system net zero and wider sustainability goals. A performance matrix model elicits outcome trade-off by assigning weights to costs, technical performance, and environmental outcomes.

**Conclusions:**

The multidisciplinary findings of this research provide valuable insight in an area where there is currently limited research, confirming and expanding on previous scoping reviews. The results highlight several comprehensive MCDA models supporting HTA sustainability framework development. Further research is warranted into the feasibility of the frameworks. Study selection and data extraction were undertaken independently due to project time constraints.

